# Editorial: Morels: physiology, genetics, and interactions with the environment

**DOI:** 10.3389/fmicb.2023.1352719

**Published:** 2024-01-08

**Authors:** Hao Tan, Xi-Hui Du, Gregory Bonito, Segula Masaphy

**Affiliations:** ^1^Sichuan Institute of Edible Fungi, Sichuan Academy of Agricultural Sciences, Chengdu, China; ^2^College of Life Sciences, Chongqing Normal University, Chongqing, China; ^3^Department of Plant Soil and Microbial Sciences, Michigan State University, East Lansing, MI, United States; ^4^Applied Mycology and Microbiology Department, MIGAL-Galilee Research Institute, and Tel Hai College, Kiryat Shmona, Israel

**Keywords:** morel, physiology, microbial ecology, mycosphere, genetics, metabolites, lifecycle, cytology

Commonly known as true morels, fruiting bodies of species of *Morchella* are important gourmet mushrooms in Ascomycota. A few species of black morels in the Elata clade of *Morchella*, such as *Morchella importuna, M. sextelata*, and *M. eximia*, and the red-blushing morel *M. rufobrunnea* in the Rufobrunnea clade, were once only wild-foraged, but have been domesticated in recent decades. Cultivation of *M. rufobrunnea* in indoor chambers was successfully reported in the United States and Israel (Ower, 1982; Masaphy, 2010). Large-scale cultivation of black morels had become feasible in China after the breeding of several high-yield varieties in combination with the development and widespread application of an appropriate organic substrate contained in the so-called exogenous nutrient bag. With a rapidly growing area, the artificial cultivation industry of black morels is expanding in China, the United States, Israel, Turkey, Canada, France, Australia, India, Japan, and several other countries.

However, morel yields are often unstable, making morel cultivation a high-profit but high-risk industry. While the practice of cultivating morels is quite empirical, our basic knowledge of morel biology is still a puzzle with many missing pieces, as summarized by Liu and Dong. Key eco-physiological and metabolic mechanisms triggering morel fruiting need more detailed understanding. *Morchella* species possess diverse ecologies, including saprotrophic and biotrophic nutrition modes. Cultivation of black morels is done in soil substrates dominated by natural microbes, rather than quasi-sterile substrates routinely used for cultivating wood-decaying fungi such as oyster mushroom and shiitake. Therefore, the growth and fruiting of morels is complex and relies on the physiological status of the morel mycelial network in combination with soil physiochemical factors and microbiota in the mycosphere. Studies are needed that combine a wide spectrum of interdisciplinary research toolkits: from fungal genetics, developmental biology, functional multi-omics, biochemistry, soil science, to microbial ecology. In this Research Topic, seven original studies expand our research toolkits to address the key problems emerged in understanding the lifecycle of morels, as well as their interactions with the environments.

To better understand the lifecycles of morels, it is necessary to monitor the nuclear changes in morel cells. *In-situ* fluorescent labeling of nuclei in living cell is one of the most useful methods. Zhang Q. et al. used an *Agrobacterium tumefaciens*-assisted transformation method to introduce a luminescent fluorescent protein into the nucleus of *M. importuna* cells, and fused the fluorescent protein with histones, so that the fluorescent protein could tightly bind to the chromosomes. Using this method, the nuclei of two monosporic strains with different mating types were labeled with green fluorescent protein and red fluorescent protein, respectively. With the help of this fluorescent labeling, independent growth of hyphae of two different mating types, as well as the stage when the two hyphae mated to form a fused nucleus, can be clearly observed. This technology will benefit the tracking the alternations between the asexual and sexual phases of morels. Genetic transformation of morels is challenging, as most stages in the morel lifecycle are in a multinucleated state, including the ascospore stage (Du et al.). The fluorescent protein labeling of the cell nucleus could be promising for capturing the mononuclear stage of morel cells in the status of living protoplasts, which is most suitable for genetic transformation.

Ascospore production is an important stage in lifecycle of morels. There is a special phenomenon whereby asexual spores can be produced in sexual reproductive structure, rarely reported for Ascomycota. Du et al. are the first to reveal that *M. galilaea* asci may have 1–16 ascospores, and there were significant differences in the shape and size of ascospores within the ascus. The *M. galilaea* ascospores can either germinate with a germination tube forming hyphae, or through directly budding at one, two or more points on the ascospores. The ascospores of *M. galilaea* possess nuclei ranging from 0 to about 20, while the conidia produced by ascospores have 1–6 nuclei. Nuclei can migrate from ascospores to conidia. This phenomenon may be related to multiple rounds of mitoses in the ascus, lack of mitosis, nuclear degeneration, or defective cytokinesis.

Multi-omic analysis is an important approach to reveal the key mechanisms involved in growth and development of morels. Fan et al. used transcriptomics and metabolomics to track three growth stages of *M. sextelata*, from hyphae to hyphae-sclerotia transition status, and to mature sclerotia. The results showed a series of differentially expressed genes and differentially accumulated metabolites, which are anticipated to play different roles at different growth stages. The differentiated functional modules provide references for understanding the mechanisms involved in transformations between different stages in lifecycle of morels. During the fruiting body stage of morels, the formation of melanin in the pileus is an important feature of black morels, which directly contributes to commercial quality of black morels. Qiu et al. revealed that blue light stimulates melanin biosynthesis in the pileus of *M. sextelata*. Structural characterization revealed that morel melanin, with a maximum absorption peak at 220 nm, contained eumelanin, pheomelanin, and allomelanin. These three components all exist regardless of the shade of the pileus color. These findings provide useful information for further determination of metabolic pathways involved in melanin biosynthesis and exploiting key genes for this bioprocess.

Morels are cultivated in complex soil environments, where soil mycosphere microbiota are crucial to the formation and development of morel ascocarps. Zhang Y. et al. reported that continuous-cropping obstacle of morels was featured by a significant decrease in the density of primordia formation, accompanied by an increase in generalist bacterial members in the soil bacterial community of continuous cropping. Continuous cropping resulted in an accumulation of nutrients in the fruiting soil bed, leading to a shift from a stochastic to a deterministic process in bacterial community assembly. Cailleau et al. reported that morel hyphae, sclerotia, and fruiting body possess divergent consortia of microbiota. Specific bacterial taxa frequently detected in association with certain structures were considered as core bacterial members. Among them, *Pseudomonas* spp. constitutes the core-associated microorganisms of morel hyphae. Confrontation experiments revealed that different strains of the associated bacteria showed either promoting or inhibitory effects on mycelium growth and sclerotia formation. Based on Koch's postulation, Zhu et al. reported that some strains of *Pseudomonas* and *Bacillus* could cause stipe abnormality in *M. sextelata*. These results demonstrated complicated interactions between mycosphere bacteria and morel hosts.

In conclusion, interweaving of findings from a diverse set of analytical approaches provides an increasingly detailed portrait of the physiology and genetics of morels and their interactions with the environment, that will further promote morel cultivation as a promising agroindustry.

## Author contributions

HT: Conceptualization, Writing – original draft, Writing – review & editing. XD: Conceptualization, Writing – review & editing. GB: Conceptualization, Writing – review & editing. SM: Conceptualization, Writing – review & editing.
